# Combination of Entecavir or Tenofovir with Pegylated Interferon-α for Long-Term Reduction in Hepatitis B Surface Antigen Levels: Simultaneous, Sequential, or Add-on Combination Therapy

**DOI:** 10.3390/ijms22031456

**Published:** 2021-02-01

**Authors:** Kanako Yoshida, Masaru Enomoto, Akihiro Tamori, Shuhei Nishiguchi, Norifumi Kawada

**Affiliations:** 1 Department of Hepatology, Graduate School of Medicine, Osaka City University, Osaka 545-8585, Japan; mykanako@gmail.com (K.Y.); atamori@med.osaka-cu.ac.jp (A.T.); kawadanori@med.osaka-cu.ac.jp (N.K.); 2 Division of Medical Science of Regional Cooperation for Liver Diseases, Graduate School of Medicine, Osaka City University, Osaka 545-8585, Japan; nishiguchi@heartfull.or.jp; 3 Department of Internal Medicine, Kano General Hospital, Osaka 531-0041, Japan

**Keywords:** chronic hepatitis B, ETV, HBsAg, HBV, peginterferon-α, TDF

## Abstract

Seroclearance of hepatitis B surface antigen (HBsAg) (“functional cure”) is the optimal endpoint of antiviral therapy for chronic hepatitis B virus (HBV) infection. Currently available anti-HBV therapy includes nucleoside/nucleotide analogs (NAs) and peginterferon-α (Peg-IFNα). Combination of NAs and Peg-IFNα, each with different mechanisms of action, is an attractive approach for treating chronic HBV infection. In earlier studies, compared with monotherapy using IFNα, combination therapy showed greater on-treatment HBV DNA suppression but no difference in the sustained response. However, responses to the combination of non-pegylated IFNα with lamivudine or adefovir were not assessed based on HBsAg quantification but were defined by normal alanine aminotransferase levels, testing negative for hepatitis B e-antigen, and low HBV DNA load over a short term. Here, we reviewed previous reports regarding the effects of combination therapy of entecavir or tenofovir with Peg-IFNα, focusing on long-term reduction in HBsAg levels. Regimens of combination therapy were classified into “simultaneous” combination (“de novo” strategy); “sequential” combination, which involved starting with one therapy followed by the other (“switch-to” strategy); “add-on” combination, which involved adding Peg-IFNα to an ongoing NAs. Some studies have shown promising results, but there is no robust evidence that combination therapy is superior to monotherapy. Large studies are needed to assess the safety and efficacy of combination therapies to increase the rates of HBsAg seroclearance over the long term.

## 1. Introduction

Hepatitis B virus (HBV) infection affects approximately 240 million people worldwide and is the major cause of cirrhosis and hepatocellular carcinoma (HCC), accounting for 686,000 deaths annually [1]. Seroclearance of hepatitis B surface antigen (HBsAg) (i.e*.,* “functional cure”) is regarded as the optimal endpoint of anti-HBV therapy to reduce HBV-related mortality because HBsAg loss is associated with improved survival and quality of life by preventing disease progression [1,2,3,4]. To reach the endpoint, several novel agents, direct-acting antivirals, and host-targeting antivirals are under clinical investigation [5]; however, unfortunately, they are not expected to be approved until a few years.

Currently available anti-HBV therapeutic drugs can be divided into two classes: nucleoside/nucleotide analogs (NAs) and immunomodulator pegylated interferon-α (Peg-IFNα). NAs efficiently suppress the pathway of HBV replication by inhibiting reverse transcription and terminating DNA chain elongation [6]. The action of NAs has little effect on the decrease in the intrahepatic replicative intermediate, a covalently closed circular DNA (cccDNA). However, increased endogenous IFN-λ3 due to treatment with a nucleotide analog may induce the expression of IFN-stimulated genes, thereby, resulting in reduced production of HBsAg [7,8,9,10]. In addition, in vitro and ex vivo studies have shown that long-term treatment with NAs could restore T-cell functions [11,12]. The major advantages of NAs include excellent safety profiles and potent antiviral activity associated with high rates of on-treatment response. Peg-IFNα has been shown to have both direct antiviral and immunomodulatory effects [6]. Treatment with IFNα can inhibit HBV replication by accelerating the degradation of pregenomic HBV RNA and decay of the core particle [13]. Moreover, it decreases the transcription of subgenomic RNA [14], which leads to a significant expansion of CD56^bright^ NK cells, accompanied by augmentation of TNF-related apoptosis-inducing ligand and IFN-γ expression [15]. The advantages of IFNα include finite course of treatment, absence of drug resistance, and durability of response post-treatment.

Theoretically, combination therapy with NAs and Peg-IFNα, each with different mechanisms of action, is an attractive approach for treating chronic HBV infection. Fundamentally, the suppression of viral replication by NAs may restore the innate and adaptive immune responses and optimize the immunomodulatory effects of IFNα for clearing infected cells. This may be due to the degradation of cccDNA by IFNα, followed by decline in the secretion of HBsAg antigen, and restoration of HBV-specific T cells, which causes further decline in intranuclear cccDNA levels. In 2013, we had reviewed previous reports of “simultaneous” [16,17] and “sequential” [18,19] combination therapies with NA and IFNα for chronic HBV infection [20]. When the combination therapy (NA and IFNα) was compared with monotherapy (IFNα), the former showed greater on-treatment viral suppression, but no difference was observed in the sustained response post-treatment. However, in almost all of these studies, responses to therapy were not assessed based on HBsAg quantification but were defined by normal alanine aminotransferase (ALT) levels, testing negative for hepatitis B e-antigen (HBeAg), and low HBV DNA load over a short term (e.g., at 24 or 48 weeks post-treatment). First generation NA (lamivudine or adefovir) in combination with non-pegylated IFNα was used in most of these studies. Since then, new generation nucleoside analogs, such as entecavir (ETV) [21,22]; nucleotide analogs, such as tenofovir disoproxil fumarate (TDF) [23] or tenofovir alafenamide (TAF) [24,25]; and Peg-IFNα [16,17] have become the first-line treatment. In addition, reports on “add-on” combination therapy have also been accumulated.

**Figure 1:**
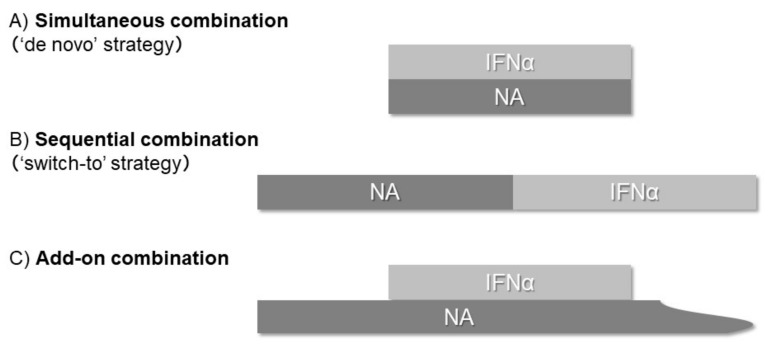
Regimens of combination therapy were classified based on the methods of drug administration: (**A**) simultaneous combination (“de novo”) strategy, (**B**) sequential combination (“switch-to”) strategy, and (**C**) add-on combination therapy.

In this review, we summarized reports regarding the effects of combination therapy of potent NAs (ETV, TDF, or TAF) with Peg-IFNα, especially focusing on the long-term reduction of HBsAg levels. Regimens of combination therapy were classified based on the methods of drug administration: simultaneous (“de novo” strategy); sequential, in which one therapy is followed by another (“switch-to” strategy); add-on, in which Peg-IFNα is added to an ongoing NAs (Figure 1).

## 2. Simultaneous (“De Novo”) Combination Strategy

Table 1 shows a summary of previous studies concerning simultaneous combination therapy for chronic HBV infection with new NAs and Peg-IFNα. These studies were grouped into ETV-based and TDF-based studies. A randomized controlled trial conducted in Thailand by enrolling HBeAg-negative patients with predominantly the genotype C showed that compared with monotherapy with 48-week Peg-IFNα, simultaneous combination therapy with 48-week ETV + Peg-IFNα did not improve the decline in HBsAg levels and HBsAg loss rate after 1 year of treatment (0.7 vs. 0.5 log_10_ IU/mL, *p* = 0.12; 9.5% vs. 4.8%, *p* = 0.49) [26]. However, Hagiwara et al. conducted a long-term, single-arm study with a mean follow-up period of 4.8 years, showing that HBeAg-positive and -negative patients with predominance of genotype C, who were treated with 48-week ETV and Peg-IFNα, had HBsAg loss rate of 3.8% after 1 year, 8.4% after 3 years, and 15% after 5 years post-treatment [27,28]. In addition, this observational study monitored changes in cccDNA levels in liver biopsies and found that the average reduction in cccDNA level was 1.4 log_10_ copies/μg after the completion of therapy.

Marcellin et al. [29] conducted an international, randomized trial in HBeAg-positive and -negative patients and demonstrated that compared with monotherapy, the simultaneous combination therapy of 48-week TDF + Peg-IFNα improved the decline in HBsAg levels and HBsAg loss after 0.5 years of treatment (1.3, 0.6, and 0.4 log_10_ IU/mL, *p* < 0.05; 9.1%, 2.8%, and 0%, *p* < 0.05 in combination, Peg-IFNα monotherapy, and TDF monotherapy, respectively). Notably, the combination therapy resulted in a particularly high rate of HBsAg loss of 35% in patients with genotype A HBV infection. The long-term follow-up of this study confirmed the results from earlier time points; the combination therapy reduced HBsAg levels by 2.4 log_10_ IU/mL and the rate of HBsAg loss by 10.4% after 1.5 years of the treatment [30]. Additionally, in a nonrandomized controlled trial in China, HBsAg loss was observed in 13% of HBeAg-positive and -negative patients in the TDF and Peg-IFNα groups after 48 weeks, whereas this was observed in only 3% patients in the Peg-IFNα group (*p* = 0.032) [31]. Here, low baseline levels of HBsAg levels after 48 weeks of treatment (odds ratio = 0.22, *p* = 0.005) was an independent factor associated with HBsAg seroclearance.

Another group from the Netherlands conducted a randomized trial on HBeAg-negative patients with HBV DNA load < 20,000 IU/mL [32], although such patients with a low HBV DNA load and no signs of necroinflammatory activity or fibrosis currently have no indicators for treatment. This study showed that simultaneous combination therapy with 48-week TDF + Peg-IFNα led to further decease in HBsAg levels (0.59 vs. 0.15 log_10_ IU/mL, *p* = 0.001) and did not improve the rate of HBsAg loss compared with the no-treatment group, after 0.5 years of therapy (4% vs. 0%, *p* = 0.38).

To summarize, it remains unclear whether simultaneous combination therapy of ETV or TDF with Peg-IFNα confers any additional benefits compared with monotherapy. The conflicting results of the two randomized trials may be attributable to the use of different NAs (ETV vs. TDF), but more likely owing to different genotype distribution: one study enrolled an Asian population with predominantly genotype C [26] and the other was an international study with all major genotypes [29]. Further studies are needed to determine the impact of simultaneous combination of ETV or TDF with Peg-IFNα for treating chronic HBV infection.

## 3. Sequential (“Switch-to”) Combination Strategy

Table 2 shows a summary of previous studies assessing sequential combination therapy for chronic HBV infection, with new NAs followed by switching to Peg-IFNα. A randomized controlled OSST trial in China [33] enrolled HBeAg-positive patients with consistent HBV DNA load ≤ 1000 copies/mL and HBeAg levels < 100 PEIU/mL after ETV treatment for 9–36 months. Following sequential combination therapy by switching to 48-week Peg-IFNα, a greater proportion of patients had HBsAg levels < 10 IU/mL and higher rates of HBsAg loss compared with those following continued treatment with ETV, at the end of treatment (15.9% vs. 0%, *p* < 0.0001; 8.5% vs. 0%, *p* = 0.003). One-year follow-up of the study indicated that sustained HBsAg loss was documented in six of seven patients with an end-of-treatment response [34]. Similarly, another randomized trial in China [35] enrolled HBeAg-positive patients who achieved HBeAg seroconversion for > 1 year with NAs (primarily ETV); the authors indicated that the discontinuation of treatment while switching to 48-week consolidation therapy with Peg-IFNα was associated with a lower risk of relapse and higher chance of HBsAg loss than consolidation therapy with NAs, during 96 weeks of post-treatment follow-up (25% vs. 58%, *p* = 0.020; 36% vs. 4.3%, *p* = 0.013).

Two controlled randomized trials in China explored the optimal duration for Peg-IFNα in sequential combination therapy. Of these, one trial enrolling HBeAg-positive and -negative patients with consistent HBV DNA load < 20 IU/mL and HBsAg levels < 2000 IU/mL after long-term treatment with an NA showed that only patients who switched to 60-week Peg-IFNα achieved HBsAg loss (32.6%) and HBsAg seroconversion (25.6%), after 1 year of treatment. However, the rates of responses did not increase following extended treatment with Peg-IFNα [36]. Another randomized New Switch trial enrolled HBeAg-positive patients who achieved HBeAg loss and HBV DNA load < 200 IU/mL following NA treatment for 1–3 years. It reported that sequential therapy with 96-week Peg-IFNα did not produce statistically higher rates of HBsAg loss compared with 48-week Peg-IFNα, 1 year post-treatment (15.3% vs. 9.8%, *p* = 0.17) [37].

In the aforementioned studies, an objective of sequential combination therapy and switching to Peg-IFNα was to prevent the relapse of hepatitis following the discontinuation of long-term NA therapy. Another objective of this strategy can be to reduce the viral load using an NA, thereby restoring sensitivity to Peg-IFNα. Notably, a randomized trial in China enrolled HBeAg-positive patients with a mean HBsAg level of 4.0 log_10_ IU/mL. This trial, however, failed to show that sequential combination therapy with ETV pretreatment for a short period (21 weeks) followed by Peg-IFNα treatment (48 weeks) improved the decline in HBsAg levels and rate of HBsAg loss compared with Peg-IFNα monotherapy (48 weeks), after 0.5 years of treatment (0.4 vs. 1.0 log_10_ IU/mL, *p* = 0.1; 1.4% vs. 4.2%, *p* = 0.3) [38].

Outcomes of the sequential combination therapy involving switching from long-term NAs (mostly ETV) to 48-week Peg-IFNα have also been reported in Japan. Tamaki et al [39] conducted a nonrandomized controlled trial in HBeAg-positive and -negative patients treated with NAs for >1 year. They showed that the decline in HBsAg levels at week 48 after the switch in the sequential combination therapy group was significantly higher than that in the matched, continued NA-treatment group (0.81 ± 1.1 vs. 0.11 ± 0.3 log_10_ IU/mL, *p* < 0.001). Regarding the factors associated with treatment response, Matsumoto et al. [40] conducted a nationwide prospective study and showed that low baseline levels of HB core-related antigen [41,42] as well as low HBsAg levels [33,37] were significant indicators of favorable outcomes after sequential therapy with NAs for ≥1 year followed by switching to Peg-IFNα. We also found that the early decline in HBsAg levels during treatment was associated with treatment response [43], as reported elsewhere [33,35,36].

Thus, sequential combination therapy, which involves switching from long-term NAs to Peg-IFNα, is considered a safe method for terminating NA therapy [4]. Although it is still unclear which patients are good candidates for sequential combination therapy, low levels of HBsAg, HBeAg, or HB core-related antigen at the switch are indicators of good response. Rapid decline of HBV antigenemia after the start of treatment is also predictive of a favorable outcome. However, termination of NA therapy always has a risk of relapse or flare-up of hepatitis; sequential combination therapy should not be indicated for patients with cirrhosis without sufficient hepatic reserve.

## 4. Add-on Combination Strategy

Table 3 shows a summary of previous studies assessing the add-on combination therapy for chronic HBV infection, in which Peg-IFNα is added to an ongoing therapy with NAs. A randomized controlled ARES trial conducted in the Netherlands [44] enrolled HBeAg-positive patients and showed that the addition of short-term treatment with Peg-IFNα (24 weeks) to a 24-week ETV treatment further reduced HBsAg levels but maintained low HBsAg loss rate after 1 year of treatment, compared with ETV monotherapy (0.8 vs. 0.4 log_10_ IU/mL, *p* < 0.001; 1.2% vs. 0%, *p* = 0.30). The long-term follow-up results of this study reported that patients in the add-on group had >1 log_10_ decline in HBsAg levels than those in the monotherapy group after over 4 years post-treatment (59% vs. 29%, *p* = 0.02); only one patient who received the add-on combination therapy reported HBsAg loss after 96 weeks [45]. Another randomized trial (PEGON) conducted in the Netherlands and China [46] enrolled HBeAg-positive patients with viral suppression by ETV or TDF for >12 months. This trial similarly showed that the addition of 48-week Peg-IFNα led to further decline in HBsAg levels after 0.5 years post-treatment compared with NA monotherapy (0.4 vs. 0.2 log_10_ IU/mL, *p* = 0.01). None of the patients showed HBsAg loss. In a post hoc analysis of two randomized trials [47] in which treatment response was defined by HBeAg loss and HBV DNA load < 200 IU/mL after 48 weeks of Peg-IFNα, response was observed in 33% and 20% patients receiving add-on combination therapy and monotherapy, respectively (*p* = 0.03). The highest response to add-on combination therapy was observed in patients with HBsAg levels < 4000 IU/mL and HBV DNA load < 50 IU/mL at randomization (70% vs. 34%; *p* = 0.01).

In addition, a randomized ANRS HB06 PEGAN trial in France among HBeAg-negative patients showed that the addition of 48-week Peg-IFNα to ongoing NA regimens for ≥1 year led to further decline in HBsAg levels but to similar rate of HBsAg loss after 2 years of treatment compared with NA monotherapy (mostly ETV or TDF) (0.88 vs. 0.30 log_10_ IU/mL, *p* = 0.004 and 8% vs. 3%, *p* = 0.15 at week 96 with add-on combination therapy and monotherapy, respectively; 1.03 vs. 0.44 log_10_ IU/mL, *p* < 0.0001 and 10% vs. 4%, *p* = 0.11 at week 144 with add-on combination therapy and monotherapy, respectively) [48].

Two nonrandomized controlled trials also yielded similar results. A multicenter study in China revealed that the addition of 48-week Peg-IFNα treatment for patients without HBeAg seroconversion during ETV therapy for ≥2 years further declined HBsAg levels but maintained low rate of HBsAg loss compared with ETV monotherapy (0.96 vs. 0.02 log_10_ IU/mL, *p* < 0.001; 4% vs. 0%, *p* = 0.49 at week 48) [49]. A multicenter study in Japan revealed that the addition of 48-week Peg-IFNα treatment in HBeAg-positive and -negative patients who received TDF maintenance therapy for ≥ 12 weeks showed a sharp decline in HBsAg by 0.2 log_10_ IU/mL/year, more often than with TDF monotherapy (41% vs. 2%, *p* < 0.001). However, none of these patients displayed HBsAg seroclearance [50]. A single-arm, observational study (HERMES) in Italy that assessed HBeAg-negative patients with genotype D HBV infection revealed that the addition of 48-week Peg-IFNα to ongoing NA regimens (mostly with ETV or TDF) for ≥ 1 year decreased HBsAg by ≥0.5-log_10_ IU/mL and ≥1-log_10_ IU/mL in 44.2% and 14.0% of patients at week 48 and 30.9% and 10.9% of patients at week 96, respectively; only one patient had HBsAg loss [51].

In summary, most studies demonstrated that the addition of a finite course of Peg-IFNα to ETV or TDF treatment resulted in further decline in HBsAg levels but maintained the low rate of HBsAg seroclearance in the short term. Fundamentally, NA treatment is continued in add-on combination therapy; thus, it is safer than sequential combination therapy that aims toward the termination of NA therapy and a drug-free state. However, it is still unknown whether the decline in HBsAg will lead to HBsAg loss. Therefore, long-term follow-up studies are needed.

## 5. Conclusions

It is still unclear whether combination therapy is superior than monotherapy for treating chronic HBV infection. Some studies concerning simultaneous, sequential, or add-on combination therapy using ETV or TDF with Peg-IFNα showed promising results. Notably, sequential combination therapy could be a safe method for terminating long-term NA therapy, although the appropriate patient candidates for the therapy remain to be elucidated. However, there is no robust evidence that combination therapy is superior to monotherapy. Although the decline in HBsAg levels was greater with combination therapy, the rates of HBsAg loss did not significantly increase in most studies. As the addition of Peg-IFNα increases cost and side effects, this strategy should be carefully assessed for individual patients, weighing all potential advantages and disadvantages. Currently, all major practice guidelines do not recommend combination therapy. Therefore, large studies are needed to assess the safety and efficacy of combination therapy to increase the rates of HBsAg seroclearance over the long term [52]. In addition, some novel direct-acting antivirals, including nucleic acid polymers and capsid assembly modulators, are under investigation in clinical trials in combination with NA and/or Peg-IFNα [53,54].

## Figures and Tables

**Table 1:** Simultaneous (de novo) combination therapy with NA and Peg-IFNα.

Author (Year)	HBeAg	*n* (Genotype, %)	Age *	Male (%)	Regimens	HBV DNA Response (%)	Seroconversion of HBeAg (%)	Decline in HBsAg (log)	HBsAg Seroclearance (%)
Tangkijvanich et al. (2016) [26]	−	63 (B16/C81)	40 + 9.8	73	48-wk ETV + Peg-IFNα	38.1% < 2000 IU/mL, 6.3% < 10 IU/mL	N.D.	−0.5	4.8%
Hagiwara et al. (2013) [27]	+/−	17 (C100)	47 + 12	76	48-wk ETV + Peg-IFNα	71% < 10,000 copies/mL at EOF	73% at EOF	−0.4	5.9%
Hagiwara et al. (2018) [28]	+/−	26 (C100)	44 + 10	69	48-wk ETV + Peg-IFNα	62% < 4.0 log copies/mL at EOF	60% at EOF	N.D.	15%
Marcellin et al. (2016) [29]	+/−	186 (A9/B27/C42/D21)	38 + 17	68	48-wk TDF + Peg-IFNα	9.1% < 15 IU/mL at wk 72	25% at wk 72	−1.3	9.1% at wk 72
Ahn et al. (2018) [30]	+/−	186 (A9/B27/C42/D21)	38 + 17	68	48-wk TDF + Peg-IFNα	24.3% < 15 IU/mL at wk 120	29.5% at wk 120	−2.4	10.4% at wk 120
Zheng et al. (2019) [31]	+/−	77 (N.D.)	30 + 7.3	71	48-wk TDF + Peg-IFNα	33.8% < 100 IU/mL at EOT	34% at EOT	N.D.	13%
de Niet et al. (2017) [32]	−	45 (A22/B7/C2/D29/E16)	43 + 12	47	48-wk TDF + Peg-IFNα	N.D.	N.D.	−0.59	4%

* Mean ± SD; EOF, end of follow up; EOT, end of treatment; ETV, entecavir; NA, nucleoside/nucleotide analogue; peg-IFNα, pegylated interferon-α; TDF, tenofovir disoproxil fumarate, N.D.; not described.

**Table 2:** Sequential combination therapy involving switching from NA to Peg-IFNα.

Author (Year)	HBeAg	*n* (Genotype, %)	Age *	Male (%)	Regimens	HBV DNA Response (%)	Seroconversion of HBeAg (%)	Decline in HBsAg (log)	HBsAg Seroclearance (%)
Ning et al. (2014) [33]	+	94 (N.D.)	33 + 8.3 *	80	>12-mo ETV →48-wk Peg-IFNα	72% < 1000 copies/mL at EOT	14.9% at EOT	−0.82	8.5
Han et al. (2016) [34]	+	62 (N.D.)	34 + 8.3 *	81	>12-mo ETV →48-wk Peg-IFNα	51.6% < 1000 copies/mL at EOT	38.7% at 1 year post-treatment	N.D.	9.7
Zhou et al. (2019) [35]	+	24 (N.D.)	35 + 7 *	67	>12-mo NA →48-wk Peg-IFNα	N.D.	27.2% at EOF	−2.2	36
Huang et al. (2017) [36]	+/−	43 (N.D.)	32 + 7.8 *	70	>2-yr NA →60-wk Peg-IFNα	N.D.	65.1% at EOF	−1.6	32.6
Hu et al. (2018) [37]	+	153 (N.D.)	35 + 10 *	82	1–3-yr NA →48-wk Peg-IFNα	34.6% < 200 IU/mL at EOF	51% at EOF	−1.09	9.8
Hu et al. (2018) [38]	+	150 (N.D.)	33 + 8.8 *	80	1–3-yr NA →96-wk Peg-IFNα	48.7% < 200 IU/mL at EOF	55% at EOF	−1.30	15.3
Xie et al. (2014) [38]	+	73 (N.D.)	30 + 8.4 *	82	21-wk ETV →48-wk Peg-IFNα	37% < 1000 copies/mL at EOF	26% at EOF	−0.4 at EOF	1.4
Tamaki et al. (2017) [39]	+/−	49 (B14/C78)	50 + 11 *	69	>12-mo NA →48-wk Peg-IFNα	78% < 2.1 log copies/mL at 48 wk	44% at EOT	−0.81	4
Matsumoto et al. (2018) [40]	+/−	95 (A7/B4/C82)	45 (27–87) ^†^	95	>12-mo NA →48-wk Peg-IFNα	N.D.	N.D.	−0.8 (inresponders)	N.D.
Enomoto et al. (2018) [43]	+/−	24 (B4/C96)	35 + 7 *	67	36–52-wk ETV →48-wk Peg-IFNα	29% < 10,000 copies at EOF	68% at EOF	−0.49 (inresponders)	8.3

* Mean ± SD; ^†^ Median (range); EOF, end of follow up; EOT, end of treatment; ETV, entecavir; NA, nucleoside/nucleotide analogue; peg-IFNα, pegylated interferon-α; TDF, tenofovir disoproxil fumarate, N.D.; not described.

**Table 3:** Add-on combination therapy that involved adding Peg-IFNα to NA.

Author (Year)	HBeAg	*n* (Genotype, %)	Age *	Male (%)	Regimens	HBV DNA Response (%)	Seroconversion of HBeAg (%)	Decline in HBsAg (log)	HBsAg Seroclearance (%)
Brouwer et al. (2015) [44]	+	85 (A5/B23/C39/D33)	32 + 10 *	74	24-wk Peg-IFNα on 24-wk ETV	77% < 200 IU/mL, 57% < 20 IU/mL at EOF	26% at EOF	−0.8	1.2
von Campenhout et al. (2019) [45]	+	48 (A8/B23/C31/D38)	33 + 11 *	73	24-wk Peg-IFNα on 24-wk ETV	69% undetectable	29%	−1.3	2.1
Chi et al. (2017) [46]	+	39 (B8/C39/D8)	35 + 9 *	72	48-wk Peg-IFNα on >12-mo ETV/TDF	77% < 20 IU/mL at EOF	21% at EOF	−0.35	0
Liem et al. (2019) [47]	+	118 (A3/B19/C38/D25)	33 + 10 *	74	24–48-wk Peg-IFNα on >24-wk ETV	85% < 2000 IU/mL, 82% < 2 00 IU/mL at EOF	24% at EOF	23% > 0.5 log decline	0.8
Bourliere et al. (2017) [48]	−	90 (N.D.)	47 (41–57) ^†^	83	48-wk Peg-IFNα on >12-mo NA	N.D.	N.D.	−1.03	10.0
Li et al. (2015) [49]	+	81 (N.D.)	32 (23–54) ^†^	62	48-wk Peg-IFNα on ETV	N.D.	48%	-0.96	4
Matsumoto et al. (2020) [50]	+/−	32 (A3/B3/C88/D3)	43 + 8 *	63	48-wk Peg-IFNα on >12-wk TDF	N.D.	43% at EOT	−0.44	0
Lampertico et al. (2018) [51]	−	70 (D100)	51 (29–64) ^†^	81.4	48-wk Peg-IFNα on NA	N.D.	N.D.	10.9% ≥ 1 log decline	1.4

* Mean ± SD; ^†^ Median (range); EOF, end of follow up; EOT, end of treatment; ETV, entecavir; NA, nucleoside/nucleotide analogue; peg-IFNα, pegylated interferon-α; TDF, tenofovir disoproxil fumarate, N.D.; not described.

## Abbreviations

ALT	Alanine aminotransferase
cccDNA	Covalently closed circular DNA
ETV	Entecavir
HBeAg	Hepatitis B e-antigen
HBsAg	Hepatitis B surface antigen
HBV	Hepatitis B virus
HCC	Hepatocellular carcinoma
NA	Nucleoside/nucleotide analogue
peg-IFNα	Pegylated interferon-α
TAF	Tenofovir alafenamide
TDF	Tenofovir disoproxil fumarate
